# 

**Published:** 1904-08-06

**Authors:** 


### NOTICE TO CORRESPONDENTS.

All MSS., Letters, Books for Review, and other matters intended for the Editor should be addressed The Editor, "The Hospital," The Hospital Buildings, 28 & 29 Southampton Street, Strand, W.C.
The Editor cannot undertake to return rejected MS., even when accompanied by stamped directed envelope.

**Notice to Advertisers and Subscribers.**

All Advertisements, Orders for Copies of The Hospital, and Business Communications should be addressed to **THE MANAGER (Not to the Editor),** The Hospital, 28 & 29 Southampton Street, Strand, London, W.C.
The Cost of Subscription, post free, is as follows:—Per week, 3½d.; per quarter, 3s. 3d.; per half-year, 6s. 6d.; per annum, 13s. Foreign and Colonial:—Per annum, 19s. 6d., payable, in all cases, in advance.

**NOTICE.—Vol. XXXV. of "The Hospital" (October 1903 to March 1904), in handsome cloth binding, is now on sale at the office of this Journal, price 10s. 6d. Cases for binding the Numbers contained in this Volume 2s. Index to Vol. XXXV., 3½d., post free.**

**The Monthly part for August is now ready. Price 1s., by post, 1s. 3d.**

### BOOKS RECEIVED.

Sharman and Co.

"The A.B.C. Instrument Check Book."

Smith, Elder and Co.

"Index of Symptoms." Third edition. By R. W. Leftwich, M.D.

University Tutorial Press.

"Matriculation Directory."

A. H. Goose.

"The Excursions of an Improvident Philosopher." By Y. H. C. P. Wanhope, M.A.

Rebman, Limited.

"Fever Nursing." By R. W. Wilcox, M.A., etc.

Cassell and Co., Limited.

"Serums, Vaccines, and Toxins in Treatment and Diagnosis."

### MEDICAL APPOINTMENTS.

John Allan, M.B., Ch.B.Edin., House Surgeon to the County Hospital, Ayr. B. E. G. Bailey, M.R.C.S., L.R.C.P., Medical Officer of the Workhouse of the Midhurst Union. G. G. Clarke, M.R.C.S., L.R.C.P., District Medical Officer of the Wakefield Union. C. W. Donald, M.D., F.R.C.S.E., Surgeon to Cumberland and Westmorland County Constabulary. E. S. B. Eames. M.R.C.S., L.R.C.P., Certifying Factory Surgeon for the Uffculme District, Devonshire. D. A. R. Farquharson, M.B., C.M.Aberd., District Medical Officer of the Chester-le-Street Union. S. G. Floyd, M.D., M.R.C.S., Certifying Factory Surgeon for the Grays District, Essex. Walter S. Frith, M.B., B.C.Cantab., Resident Medical Officer to Felstead School and Head of the Junior House. E. W. Longden, L.R.C.P., and S.Edin., L.F.P.S.Glasg., District Medical Officer of the Luton Union. W. Leslie Lyall, M.B., C.M., reappointed non-resident Clinical Assistant to the Victoria Hospital for Consumption, Edinburgh. David P. Marais, M.B., Ch.B., Resident Physician to the Victoria Hospital for Consumption, Edinburgh. William H. Maxwell, M.A., M.B.Camb., F.R.C.S.Eng., Senior Resident Medical Officer to the London Temperance Hospital, Hampstead Road, N.W. Adam L. H. Oakley, L.R.C.P.Edin., Third Honorary Anæsthetist to the London Throat Hospital, Great Portland Street. G. L. S. A. Roper, District Medical Officer of the Chesterton Union. C. Todd, M.D.Cantab., Director of the Serum Institute of the Egyptian Sanitary Service, Cairo, Egypt. F. D. Turner, M.B.Lond., M.R.C.S., L.R.C.P., Medical Officer to the Post Office and Medical Examiner to the Board of Education at Huddersfield.

**"The Hospital" Institutional Library.**

**"Hospitals and Asylums of the World." 4 Vols., with a Portfolio of Plans, complete £12 12s.**
**"Burdett's Hospitals and Charities, 1904." 5s. net.**
**"Burdett's Official Nursing Directory." 3s. net.**
**"Cottage Hospitals, General, Fever, and Convalescent." 10s. 6d.**
**"Uniform System of Accounts for Hospitals and Public Institutions." New Edition. 4s. net.**
**"Hospital Expenditure: The Commissariat." 2s. 6d.**
**"A Legal Handbook for the Use of Hospital Authorities." 2s. 6d.**
**"The Cottage Hospital Case Book or Register of Patients." 10s. and 12s. 6d.**
**All these are published by the SCIENTIFIC PRESS, Ltd., and may be obtained through any bookseller, or direct from the publishers, 28 and 29 Southampton Street, Strand, London, W.C.**

A MOST VALUABLE
HELP TO PRIVATE NURSES.

### THE CASE-BOOK FOR PRIVATE NURSING.

Arranged for Day and Night Nurse's Report, with rulings in accordance with what has been found in practice to be the most suitable and approved System for Private Nurses, &c. Size of Book, $7\frac{1}{2}$ inches by $9\frac{1}{2}$ inches, containing 48 pp.,

AT THE PRICE OF AN ORDINARY EXERCISE BOOK.

**3d.**, by post **$4\frac{1}{2}$d.**

SPECIAL PRICE FOR A DOZEN OR MORE COPIES.

THE SCIENTIFIC PRESS, LTD.,
**Publishers of Nursing Text-Books.**
28 & 29 Southampton Street, Strand, London, W.C.
**Complete Catalogue Post Free on Application.**

WE HAVE JUST PUBLISHED WHAT WE BELIEVE TO BE A BOON TO THE BUSY NURSE

***AT A PRICE THAT WILL INTEREST YOU.***

### THE POCKET-BOOK OF TEMPERATURE CHARTS

Containing **17** Twelve-Hour and **8** Four-Hour Charts, $7\frac{1}{4}$ inches by 5, bound up together in book form and perforated so that each Chart may be removed after use.

Price **SIXPENCE**, by post 7d.

*The Nurses' Journal* says:—"We consider it a **most valuable companion** to the 'Case-Book for Private Nursing.'"

THE SCIENTIFIC PRESS, LIMITED,
**28 & 29 Southampton Street, Strand, London, W.C.,**
*AND OF ALL BOOKSELLERS.*

